# Microfluidic Determination of Cell-Derived ATP and Single Cell Pressure Mapping Confirms Benefits of Normoglycemic Stored Red Blood Cells

**DOI:** 10.1021/acsmeasuresciau.5c00032

**Published:** 2025-07-03

**Authors:** Stephen A. Branch, Yunong Wang, Samuel Azibere, Logan D. Soule, Ashley R. Davis, Timothy McMahon, R. Scott Martin, Lane A. Baker, Morgan K. Geiger, Dana M. Spence

**Affiliations:** Departments of Biomedical Engineering and Institute for Quantitative Health Science and Engineering, 3078 Michigan State University, East Lansing, Michigan 48824, United States; § Department of Medicine, 609772 Duke University, Durham, North Carolina 27710, United States; ∥ Department of Chemistry, 7547 Saint Louis University, St. Louis, Missouri 63103, United States; ⊥ Department of Chemistry, Texas A & M University, College Station, Texas 77843, United States

**Keywords:** RBCs, normoglycemic, automated, storage solution, oxidative stress, storage lesion

## Abstract

In the United States, ∼30,000 units of red blood cells (RBCs) are transfused daily to patient recipients. These RBCs are stored in one of multiple variations of media known as additive solutions, all of which contain glucose at concentrations well above physiological levels. Recently, strategies for storage of the RBCs in normoglycemic versions of the additive solutions whose glucose levels are maintained with periodic boluses of glucose were developed, resulting in benefits to the stored RBCs. Here, we describe a system capable of semiautonomous, Wi-Fi-enabled control of glucose delivery using a microperistaltic pump for maintenance of physiological concentrations of glucose in a closed RBC storage system. The RBCs stored in these normoglycemic conditions demonstrated reduced lysis and reduced hemoglobin glycation in comparison to those of the currently used hyperglycemic additive solutions. Furthermore, a novel single cell technique using pressure-induced conductivity mapping showed an improved Young’s modulus for those RBCs stored in normoglycemic solutions. These quantitative measurements of the RBCs’ chemical and physical properties coincide with improvements in cell functionality. Specifically, determinations of RBC-derived ATP using a 3D-printed microfluidic device show an increased release of ATP for RBCs stored in normoglycemic solutions in comparison to hyperglycemic storage, even for cells that were 2 weeks past a storage expiration of 42 days.

## Graphical abstract

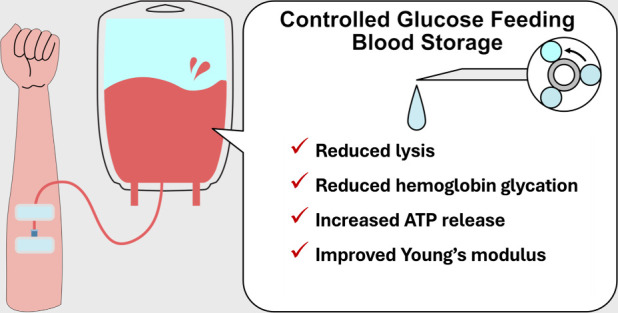

## Introduction

The first successful human-to-human blood transfusion was administered by James Blundell in 1825, marking an important milestone in the beginning of a critical field of modern medicine.^1^ Now, nearly 200 years later, the most recent available National Blood Collection and Utilization Survey (from 2021) estimated that more than 10.5 million units of red blood cells (RBCs) are transfused every year in the United States alone.^2^ From the management of anemias to trauma stabilization, the availability of high quality blood products is key to proper patient care.

Notably, virtually all contemporary RBC transfusions continue to use donor-derived cells.^3^ Thus, donation, and the subsequent processing and storage, of RBCs remains the only viable option to maintain the supply of these life-saving products.^3–5^ In the United States, the Food and Drug Administration (FDA) now regulates every step of blood drawing, processing, storage, and transport.^6–8^ These steps involve removal of ∼450–500 mL of whole blood from a donor drawn into a bag prefilled with an anticoagulant to prevent clotting and subsequent spoilage.^9,10^ Following the separation of the RBCs from the other whole blood components, the RBCs are mixed into a storage bag with prefilled additive solution that helps keep them healthy throughout the storage period.^9,10^

Of key importance are the anticoagulant and additive solutions used for RBC storage. Prior to the development of these solutions, first started in 1915 by Francis Rous and J.R. Turner, blood could not be stored before transfusion; donated blood had to be used immediately.^9^ In addition to enabling blood storage, these solutions contribute to the mitigation of storage-induced degradations in RBC health that impair transfusion efficacy.^9^ These degradations, collectively termed the storage lesion, are chemical and physical changes that occur to the RBC during storage. Currently, there are multiple collections and additive solutions used during the processing of whole blood and storage of the RBCs. Previously, we reported that collection of whole blood and storage of RBCs in normoglycemic solutions, as opposed to the hyperglycemic levels found in current collection and additive solutions, resulted in beneficial effects for the stored RBC.^11–13^

We recently reported on advancing those normoglycemic storage strategies with a semiautomated, valve-controlled delivery system for maintenance of glucose concentrations in the stored cells^14^ using a reservoir of feeding solution connected to a solenoid valve and suspended above the red blood cell (RBC) storage bags. This first-generation feeding system worked well throughout multiple RBC storage periods. However, several drawbacks exist that may have impacted system performance and translational potential. First, the feeding solution was gravity-fed, and therefore, flow was dependent on the relative elevation of the feeding solution reservoir. Another drawback of our previously reported feeding system was that the valves necessitated direct contact with the feeding solution. While care was taken to clean these valves, they could not be autoclaved or easily disassembled for sterilization. While it is worth noting that blood agar swabs following initial storage trials showed no signs of bacterial contamination, future clinical approvals using this system could prove to be challenging. Finally, the previous system did not individually manage each storage bag. A general feeding schedule was established with delivery volumes scaled based on the initial bag volume and values of known glucose consumption by the stored RBCs as a function of time. This strategy accounts for increases in volume due to feeding but not reductions due to sampling for experimentation. It also does not allow for glucose feedback, preventing the system from compensating in the case of hypoglycemia or hyperglycemia. Since certain values were not accounted for during the storage duration, separate systems were required for each set of blood bags in storage.

Here, we report on a next-generation system that enables wireless control of remote administration of glucose maintenance in a parallel format. Use of peristaltic pumps (instead of gravity-based feeding through valves) coupled with an improved dosing algorithm has led to improvements in experimental control, rigor, and flexibility as well as system usability and translational potential. Importantly, we use several analytical measurements to confirm these improvements to the overall stored RBC health. This includes a novel, SICM-based single cell pressure mapping of individual cells, as well as bulk measurements of the RBC’s ability to release ATP using 3D-printed microfluidic technologies.

## Materials and Methods

### Blood Collection, Separation, and Storage

All blood was collected via forearm venipuncture from informed and consented donors following procedures approved by the Biomedical and Health Institutional Review Board at Michigan State University. Approximately 7 mL of whole blood were collected into untreated (no commercially added anticoagulant) 10 mL glass Vacutainer tubes (BD; Franklin Lakes, NJ) containing 1 mL of in-house made CPD (a citrate, phosphate, dextrose solution currently used in blood storage strategies) or CPD-N (a solution that is the same as the CPD but with normoglycemic concentrations of glucose) anticoagulants sterilized via autoclave at 121 °C and 21 bar for 30 min. Table 1 describes the contents of the collection and subsequent additive solutions (AS-1 and AS-1N) used in these studies. Six tubes (each containing the aforementioned ∼7 mL of whole blood) of each condition were collected from all donors and allowed to sit at room temperature for 30 min with inversion of the tubes every 5 min. Blood components were then separated by centrifugation at 2000*g* for 10 min, followed by aspiration of the plasma, buffy coat, and top 2 mm of packed RBCs (pRBCs). For each donor, the remaining pRBCs were pooled by condition in a 50 mL conical tube and added in a 2:1 ratio to either AS-1 or AS-1N additive solutions, likewise sterilized by autoclave. These RBCs were then transferred into sterile 100 mL veterinary blood storage bags (ABRI catalog no. BG-DD 100 BAG). All steps following centrifugation took place in a biosafety cabinet to maintain sterility.

**Table 1:** Components of CPD and AS1 and Modified Normoglycemic Versions

	Component			
	CPD	CPD-N	AS-1	AS-1N
Trisodium citrate (mM)	89	89		
Citric acid (mM)	16	16		
Monosodium phosphate (mM)	16	16		
Dextrose (mM)	129	5.5	111	5.5
Sodium chloride (mM)			154	154
Adenine (mM)			2	2
Mannitol (mM)			41	41
pH	5.6	5.6	5.8	5.8
Osmolarity (mOsm L^–1^)	533	410	465	357

### Use of a Semi-Automated, Remote Controllable Feeding System

A partial description for glucose maintenance of the RBCs in storage is shown in Figure 1. An open-source single-board computer (SBC) (Orange Pi Zero2; Shenzhen Xunlong Software Co., Ltd.; Shenzhen, China) was used as the control server for the feeding system (Figure 1A). In addition to enabling operation of the feeding software, the SBC also acts as a Wi-Fi host to which client devices can connect. Client devices are redirected to the web interface of the software upon opening a web browser and navigating to any Web site via domain name system redirection. The feeding software, written entirely in the Python programming language, is described in detail in the Supporting Information. The microcontroller board drives up to four stepper motors with peristaltic pump heads. These allow for closed-system pumping without direct contact with the working fluid. Thus, a reservoir of glucose feeding solution can be pumped into RBC-containing storage bags while maintaining sterility. The stepper motors used to drive the peristaltic pump heads can be controlled with high precision, enabling rotation in 0.225° increments and volumes as low as 160 nL, although the precision of the delivered volume increases as the bolus volume increases. Early on in glucose storage, when glucose consumption was at a higher rate that later in storage, a typical bolus was between 800 and 1,000 nL every 10–15 min. Near the end of storage, the frequency of such a bolus was several hours. The RBCs were mixed daily by gently massaging the storage bag containing the cells.

**Figure 1:**
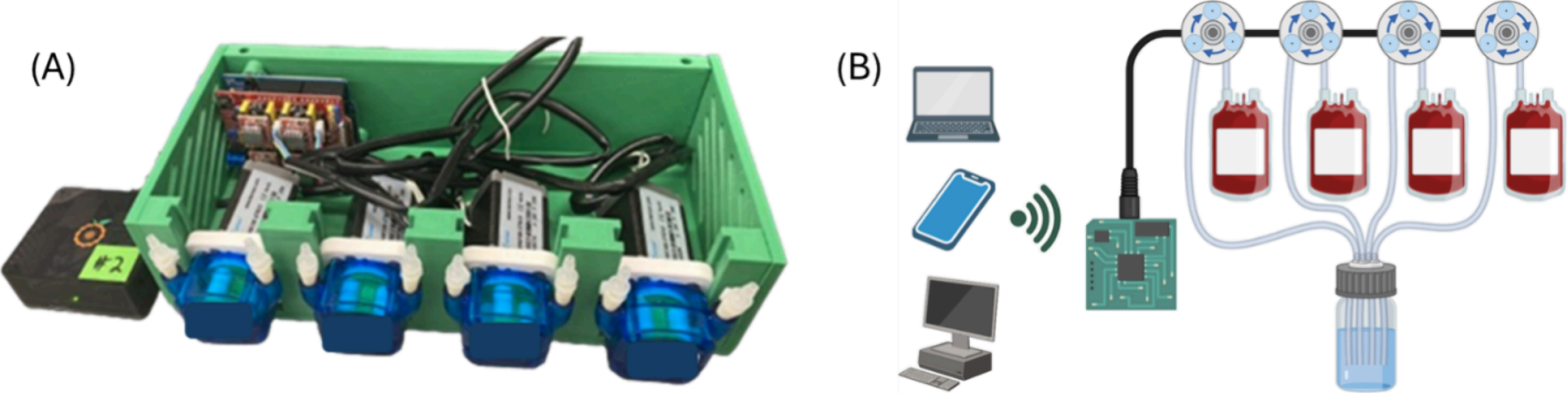
Assembled pumping system with a control server and stepper motors controlling peristaltic pumping is shown in (A). The two connection ports on each of the 4 pumps on the front of the assembled system are used to connect to the RBC storage bags for glucose delivery. A graphical overview of the Wi-Fi-enabled semiautomated RBC feeding system is shown in (B). The directional arrows represent the pumps controlled by stepper motors. The feeding software, written in-house, is described in the Supporting Information.

RBCs undergoing normoglycemic storage were then connected to the glucose maintenance system, as shown in Figure 1B. All tubing used in the system to maintain glucose concentrations was UV sterilized and/or chemically disinfected using a 10% bleach solution followed by a thorough flush using 18.2 MΩ·cm water. Sterilized 1.5 in., 18-gauge blunt tip needles were connected to the tubing, and the entire system was primed with feeding solution. This solution contained 300 mM glucose in 18.2 MΩ·cm water and was sterile filtered using a 0.22 μm pore Stericup (MilliporeSigma; Burlington, MA). The needle was used to pierce the spike port and inserted fully into the bag, forming a tight seal between the port collar and the needle base. All RBC units were kept in cold storage at 2 – 6 °C for 6 to 8 weeks. The design algorithm for maintenance of glucose concentrations within the storage bags is shown in the Supporting Information as Figure S1, along with a detailed description algorithm.

### Sampling of RBCs for Experimentation

Stored RBCs were sampled periodically to monitor glucose concentrations in the bags and to perform various experiments to determine the RBC chemical and physical properties. A 1 mL syringe was used to remove aliquots of RBCs from each bag in storage via a sampling port. The graduations on the syringe were used to estimate the volume of each aliquot and were entered into the feeding system shown in Figure 1A to maintain an accurate bag volume for feeding solution dosing. Determination of glucose concentrations in the storage bag generally required only a few microliters to be removed, while weekly experiments to determine other RBC properties (described below) required approximately 300 – 400 μL.

### Glucose, HbA1c, and Hemolysis Measurements

Glucose measurements were performed approximately every 3 days to provide information to the feeding system. A commercial glucometer (AimStrip Plus; Germain Laboratories; San Antonio, TX) was used to measure glucose levels in the RBC-containing storage bags. Hematocrit measurements were performed using a hematocrit centrifuge and a microcapillary reader (StatSpin; HemoCue America; Brea, CA). RBC sample pH measurements were performed with a micro pH electrode (STMICRO5; OHAUS Corporation; Parsippany, NJ). The ratio of glycated hemoglobin (HbA1c) in the RBCs was measured using an FDA-approved analyzer (DCA Vantage; Siemens Healthineers; Erlangen, Germany). The degree of lysis in the storage bags was measured with absorbance spectroscopy by using a microplate reader (SpectraMax M4; Molecular Devices; San Jose, CA). An aliquot of stored RBCs was centrifuged at 2000*g* for 15 min. Approximately 80 μL of supernatant and 20 μL of the RBC pellet were collected. The supernatant was diluted 10-fold and the packed RBC pellet was diluted 1000-fold in Drabkin’s reagent (Sigma-Aldrich; St. Louis, MO). Absorbance was measured at 540 nm and quantified against six standards prepared from lyophilized human hemoglobin (Sigma-Aldrich; St. Louis, MO) reconstituted and diluted in Drabkin’s reagent to concentrations between 0–1 mg/mL. The lysis percentage can then be calculated using the following formula:

1 $$\mathrm{Lysis}\;\%=\frac{\left[Hb_{\mathrm{supernatant}}\right]\left(1-\mathrm{HCT}\right)}{\left[Hb_{\mathrm{supernatant}}\right]+\left[Hb_{\mathrm{cell pellet}}\right]}\times 100$$

### Determination of ATP Release

A 3D-printed device was used in this study for detecting ATP from RBCs and was based on a previous design,^15^ optimized for nitric oxide (NO) and ATP detection from flowing RBCs. The NO portion of the device was not used in this work. The device was designed in Autodesk Inventor Professional and printed on a Stratasys J750 PolyJet printer with Vero UltraClear model material. The device was manually cleaned of support material and rinsed with isopropanol and water, with any small ridges forming a carpet layer on the bottom of the device (from the support material) removed by wet polishing until the device was smooth and transparent. All channels were designed with a 500 × 500 μm cross-section, and fluidic connections were made with printed threads designed to accommodate commercial fittings (Idex P-202 IDEX Health & Science, Oak Harbor, WA) and tubing (508 μm I.D. Tygon tubing, 06419-01, Cole-Parmer. The previously described device^16^ had ports for introduction of electrodes (for NO detection) that were not used in this study. These ports were blocked by threading into the device various prefabricated and commercially available electrodes to make the device complete and possibly prevent it from leaking prior to ATP detection. The chemiluminescence detection part of the device is the significant component for this study. It consists of two inlets for introducing the luciferin/luciferase solution, followed by a straight reaction channel. The optimized design resulted in reagent streams that converge with the sample channel to form a double-mixing T, and the light-producing reaction occurs as the solutions mix via diffusion in the ATP detection channel.

In this study, phosphate-buffered saline (PBS) was used as the carrier buffer and pumped through the device at 15 μL/min from a syringe pump. Injections were made using a 4-port injector fitted with a 1 μL volume rotor (Valco Instruments, Houston, TX). The ATP/luciferin/luciferase reaction channel was placed over a photomultiplier tube (Hamamatsu Photonics) in a light-excluding black box. The luciferin/luciferase solution contained 10 mg/mL crude firefly lantern extract (Sigma-Aldrich, St. Louis, MO), and 1 mg/mL luciferin (Gold Biotechnology, St. Louis, MO) in PBS. The solution was sterile filtered, loaded into two syringes, and continuously pumped into the device at 2.5 μL/min.

For long-term RBC storage studies, all solutions were prepared fresh on the day of the experiment. An ATP stock solution of 25 μM was prepared in PBS. The freshly isolated RBCs that were suspended in a storage solution (at ∼60% hematocrit) were diluted with PBS to a final volume of 1.25 mL and a final hematocrit of 7%. Standard addition curves were created using 1 mL of RBCs and 0–250 μL of ATP stock solution. Both RBC samples were spiked with standard ATP solutions immediately before 1 μL plugs of the standard addition samples were injected into the device via a 4-port injector (with a 150 μm i.d. fused silica capillary connecting the 4-port to the device). The injected RBC samples resulted in signals represented by chemiluminescent peaks. The peak heights from ATP-spiked samples, injected in triplicate, were used to determine ATP concentrations from RBCs stored in the various storage solutions (AS-1 vs AS-1N). The method of standard addition was used to quantitate the ATP for all of the RBC studies to account for any matrix effects. All data was smoothed in PeakFit (San Jose, CA) with a Savitzky-Golay filter (0.5% window) to filter noise for analysis and presentation purposes.

### Single-Cell Pressure Mapping

For pressure mapping studies, the RBC samples were diluted to a hematocrit of ∼0.7% and centrifuged. The supernatant was removed and replaced with either fresh additive solution or a physiological salt solution (PSS).^11^ After mixing by vortex, the RBC sample was transferred onto a poly-l-lysine coated glass slide (Poly-Prep Slides, Sigma-Aldrich) for cell attachment for 30 min prior to scanning ion conductance microscopy (SICM) imaging. The SICM was set up over an inverted optical microscope (Eclipse TE2000-U with DMK 37BUR0234 CMOS sensor, The Imaging Source), to facilitate the positioning of the nanopipette and obtain bright-field images of the RBCs. The pipet of ∼200 nm I.D. tip size on average was fabricated using a heat-filament puller (P-1000, Sutter Instruments) with a borosilicate single-barrel capillary (1.0 mm O.D., 0.58 mm I.D., 10 cm in length, Sutter Instruments) using the following parameter: HEAT = 505, PULL = 65, VEL = 78, DELAY = 167, PRESSURE = 500, with the RAMP = 505 using Delay Mode. The optical microscope was placed over an antivibrational stage (AVI-200S/LP, Herzan). Pipette movement was achieved with a *Z*-axis stepper motor (M-112.1DG1, Physik Instrumente), a *Z*-axis piezoelectric actuator (P-753.21C, Physik Instrumente) and an *XY*-axis piezoelectric actuator (P-621.2CL, Physik Instrumente). Nanopipettes were pressurized through the high-pressure gas line controlled through a pressure valve (TM-200, Narishige) with a custom-designed pipet holder. The SICM potentiostat (Axopatch 200B, Axon Instrument) was connected to the pipet to apply voltage and measure ion current. A multichannel oscilloscope (Axon Digidata 1550B, Axon Instrument) was connected to a computer for monitoring all of the signal channels. An FPGA board (NI-7845R OEM, National Instruments) was programmed to communicate with a home-built LabVIEW (2024, National Instruments) and Python (version 3.9.5) hybrid-designed software on the computer. The pipet was back-pressurized with 2 kPa and brought ∼12.5 μm away from the sample surface first, which is within the reach of the *Z*-axis piezo actuator. Before scanning, the probe hopped at the same XY position 200 times to obtain 200 ion current approach curves as the substrate reference. Then, the probe raster scanned the RBC region of interest with a pipet approach rate of 7 μm/s and retract rate of 35 μm/s with 5 μm hopping height and 2.1% ion current threshold. During the pipet approach at each XY pixel, ion current and piezo movement traces between 1% and 2% threshold were recorded and saved as data files.

During the AS-1 and AS-1N experiments, samples were imaged with the original buffer first (AS-1/AS-1N) and then the buffer was replaced with PSS, and the same region was imaged again. Approach curves were extracted and analyzed by using custom-built Python scripts. RBC topography was obtained by extracting the piezo final location when the ion current reached a 2.1% threshold at each XY pixel. Linear regression was performed on 200 substrate-referential approach curves and on RBC scanning approach curves. Substrate referential I-Z curve slope, *s*_*∞*_, was chosen by picking the median value of all the slopes of substrate-referential approach curves. The Young’s modulus at each pixel was obtained using the equation reported by Rheinlaender et al.^17^ For statistical analysis, only the pixels over the cells were extracted by applying the Watershed imaging segmentation method (using the scikit-image Python library) to the topography to isolate the location of red blood cells. Young’s moduli pixelwise histograms were obtained by summarizing all pixels from categories: fresh cells (in PSS), AS-1 cells (in AS-1), AS-1 cells (in PSS), AS-1N cells (in AS-1N), and AS-1N cells (in PSS). These cells were measured during week 6 of storage.

## Results and Discussion

### Glucose Maintenance and Resultant Cell Lysis

Improvements in blood storage have come about primarily through the development of improved additive solutions.^18^ These solutions help to preserve RBCs throughout storage by providing them with nutrients and enable easier transfusion by reducing the viscosity of stored RBCs.^9^ However, current FDA-approved additive solutions contain extreme amounts of glucose (up to 111 mM), which have been implicated in the development the storage lesion.^11–13,19^ Red blood cell storage using experimental normoglycemic additive solutions shows promise in mitigating components of this storage lesion by improving RBC deformability and ATP release and reducing oxidative stress and osmotic fragility.^11–14,19^ Here, a semiautonomous, remotely controlled feeding system for normoglycemic RBC storage was developed. This iteration improves upon previously published work^14^ to enable better experimental control, allowing for more rigorous study of low-glucose blood storage.

While a previous feeding system^14^ enabled acceptable glucose control throughout the 42-day storage period, the system reported here enables semiautomated glucose correction and higher volumetric precision compared to previous systems. This current system (Figure 1) was used to deliver varying volumes of 300 nM glucose to storage bags containing RBCs from n = 4 donors. Using a target glucose concentration of 5 ± 1 mM in the AS-1N storage bag, the system was able to maintain that range of glucose in 48 of 64 measurements performed over a 56 day storage period (Figure 2A). No glucose was added to the AS-1 stored cells due to the concentration in those bags ranging from slightly below 50 mM on day 1 and never decreasing below 35 mM for any of the donor samples.

**Figure 2:**
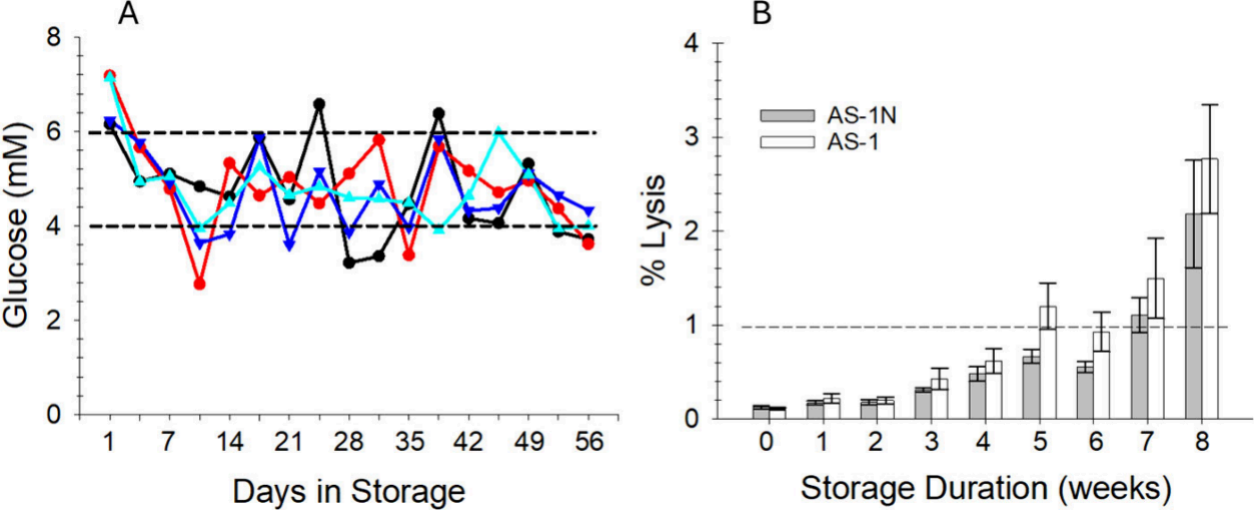
Concentrations of glucose for *n* = 4 donors across 56 days of storage in AS-1N is shown in (A). Of the 68 measurements shown, 48/64 (75%) are within the range of 5 ± 1 mM. The glucose levels were maintained by measuring the glucose concentration of an aliquot removed from the storage bag, followed by the addition of an appropriate volume of a sterile glucose stock solution using the system described in Figure 1. The percentage of lysed RBCs within the storage bag is shown in (B). Importantly, the lysis rate for the AS1-N stored RBCs remained under 1% (required by the American Association of Blood Bankers, AABB) up through 6 weeks of storage; however, both storage methods resulted in lysis rates above 1% beyond 6 weeks of storage. For both measurements, *n* = 4 donors, and error bars represent SEM.

RBC lysis was also measured for cells stored in a traditional hyperglycemic AS-1 additive solution and compared to AS-1N storage with periodic feeding. As shown in Figure 2B, there was no significant difference in cell lysis between the two storage solution strategies and both AS-1 and AS-1N were below the 1% lysis threshold set forth by the FDA through 4 weeks of storage. However, there was a significant difference in RBC lysis that was dependent on storage solution after 5 and 6 weeks of storage duration. Specifically, while the AS-1 storage strategy was not statistically different than the 1% threshold, the RBCs stored in AS-1N were statistically less than 1% (*t* = 0.05 for both tests). Furthermore, at 7 weeks of storage (which is 1 week past normal storage duration allowed in the United States) the AS-1N lysis percentage was not statistically different than 1% (*t* = 0.05). While AS-1N may not improve RBC lysis enough to extend storage durations, decreases in hemolysis relative to traditional hyperglycemic storage near current storage end points may be clinically relevant. Coupled with our previous reports of reduced osmotic fragility of RBCs stored in AS-1N solutions with periodic feeding,^14^ overall improvements in post-transfusion RBC circulation may be attainable with normoglycemic storage.

### Significant Changes in Glycated Hemoglobin within the Stored RBCs

Glycated hemoglobin (HbA1c) levels in stored RBCs likely increase throughout storage due to the hyperglycemic conditions present in current additive solutions, and such measurements have been reported.^20–23^ While glycation may play a role in the increased fragility and impaired post-transfusion function of these RBCs, these prior studies were in agreement that the small increases in HbA1c were most likely not clinically significant. However, these reports investigated only RBCs stored in hyperglycemic environments. The data in Figure 3 provide evidence that normoglycemic storage of the RBCs in AS-1N was beneficial in controlling the increase in HbA1c versus RBCs stored in AS-1. While the conventional, hyperglycemic AS-1 additive solutions did not result in a significant positive increase in HbA1c until week 6 of storage, there was a significant difference in the HbA1c between AS-1 and its normoglycemic counterpart, AS-1N even at 28 days in storage. Thus, normoglycemic storage may prevent glycation of RBC proteins (in addition to hemoglobin) and formation of early or advanced glycation end-products. This may help alleviate post-transfusion complications, including endothelial damage, inflammation, and lung injury.

**Figure 3:**
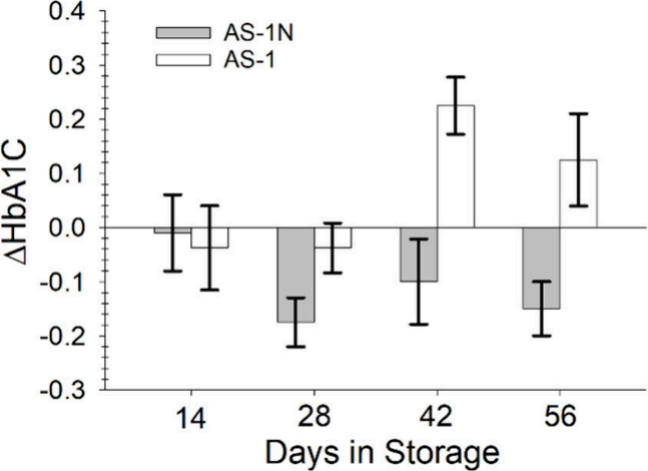
Changes in HbA1c for normoglycemic storage in AS-1N (gray bars) and AS-1 (white bars). Changes in HbA1c were measured as the difference between the HbA1c value on day 1 of storage and days 14, 28, 42, and 56 for each of the samples. There is a significant difference in the HbA1c values between AS-1N and AS-1 for each day past 14 days of storage (*t* = 0.05). The error bars represent the SEM of either *n* = 8 (days 14, 28, and 42) or *n* = 4 (day 56) donor samples.

### Improvements of RBC-Derived ATP

RBCs act as important regulators of vascular tone by releasing ATP in response to various stimuli hypoxia or shear stress on the cell caused by flow-induced mechanical deformation.^24–27^ This free ATP acts as a purinergic messenger, causing vasodilation via the synthesis of nitric oxide in the endothelium.^15,28^

However, hyperglycemic storage of RBCs has previously been shown to significantly reduce flow-induced ATP release.^11–13,29^ However, to date, measurements of RBC-derived ATP in stored cells have never been measured beyond 6 weeks of storage.

Here, a 3D-printed microfluidic device (Figures 4A and 4B) was employed to determine the concentrations of ATP from RBC samples that were stored in AS-1 and AS-1N over the course of 8 weeks (2 weeks beyond normal expiration of stored cells) using the method of standard additions. RBC samples were pumped through the inlet of the device at 15 μL/min, and luciferin/luciferase was pumped from two syringes at a rate of 2.5 μL/min (to each side channel). Analysis of ATP in the RBC samples was performed by injecting 1 μL plugs of the standard addition samples into the device. This device has been previously characterized in terms of mixing and sensitivity, with a 30 nM ATP limit of detection.^15^ In these studies, a 3D-printed device was used to quantitate the concentrations of RBC-derived ATP in both AS-1 and AS-1N RBC samples. Prior to injection into the device, the RBCs were diluted to 7% hematocrit. While it is established that ATP release from RBCs under control experiments vary depending on the method used, hematocrit measured, and RBC source, acceptable values are generally in the hundreds of nanomolar range.^11^ As shown in Figure 4C, ATP levels from RBCs stored in AS-1N were found to be fairly consistent throughout the weeks of storage. In contrast, ATP levels from RBCs stored in AS-1 decreased over time, which is consistent with the results of previous reports.^11–13,29^

**Figure 4:**
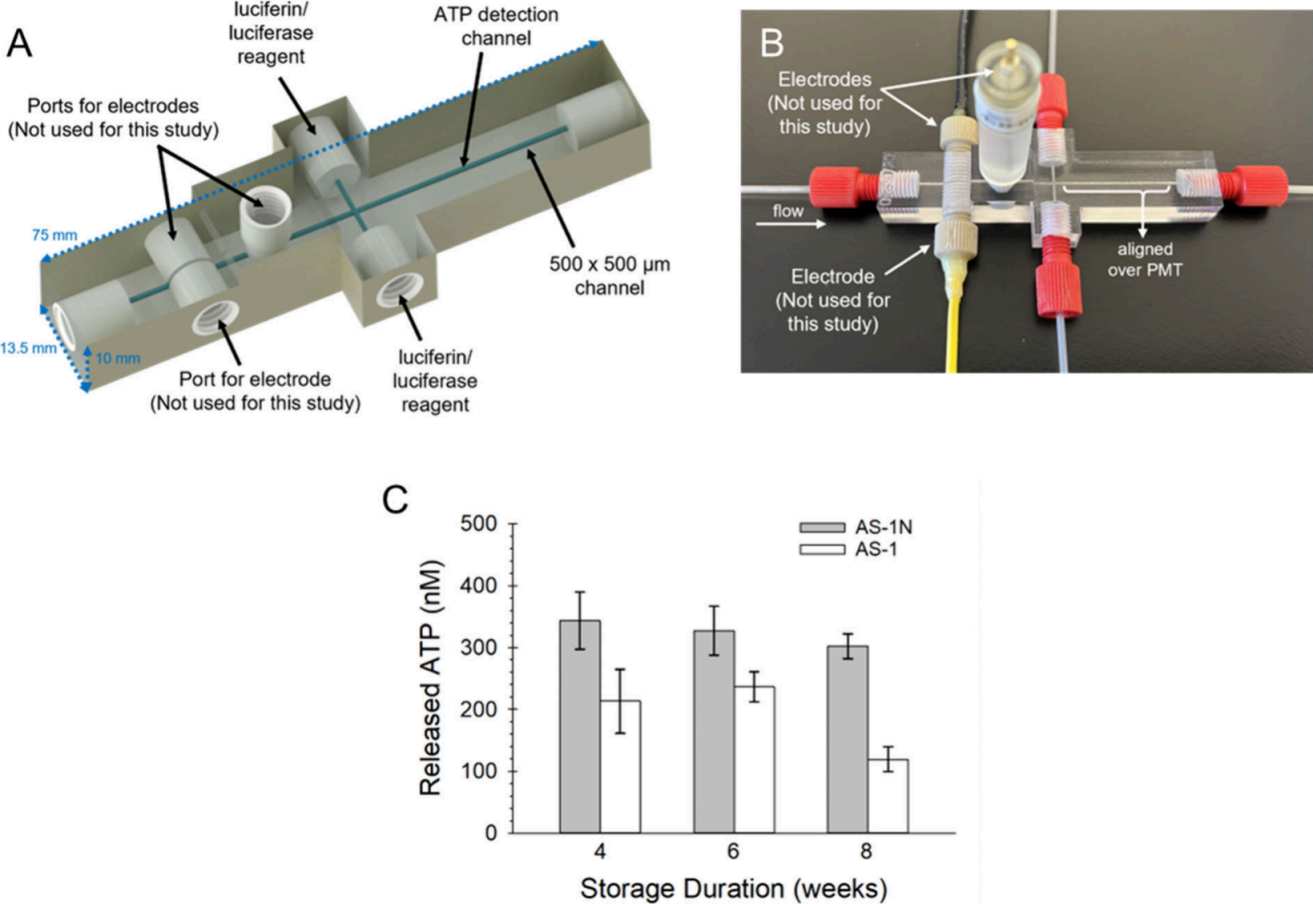
(A) CAD rendering of the 3D-printed microfluidic device used in this study. The device contains threaded ports for fluidic fittings and electrodes. (B) The assembled device with threaded electrodes for amperometric detections was not employed in this study. ATP mixing channel is aligned directly on top of a PMT window for chemiluminescence detection. (C) RBC-derived ATP was measured under flow conditions for RBCs stored in AS-1N (gray bars) and AS-1 (white bars). The RBCs stored in the AS-1N released significantly higher amounts of ATP than the cells stored in AS-1 (*t* = 0.05). Importantly, the ATP release from the RBCs stored in AS-1N continued to release ATP at levels similar to those measured on day 1 of storage past typical expiration dates of 6 weeks. The data are averages of 4 donor samples, and error bars are SEM.

The improvements in flow-induced ATP release from RBCs stored under normoglycemic conditions could have important implications *in vivo*. Previously, we reported an increase in nitric oxide production from bovine pulmonary endothelial cells using a microfluidic chip consisting of stored RBCs flowing under inserts containing the cultured endothelium.^11^ The ability of RBCs to regulate vasodilation through this mechanism could be impaired by hyperglycemic storage but maintained using a normoglycemic anticoagulant and additive solutions.

### Single Cell Pressure Mapping of Stored RBCs

The ability of RBCs to deform as they pass through the microvasculature and splenic pulp is critical.^30^ Deformable cells are less prone to sublethal membrane damage and sequestration within the spleen, improving circulation time, and contribute to healthy hemorheology and tissue oxygenation.^31^ This shear-induced deformation also results in the release of intracellular stores of ATP into the bloodstream, helping regulate vasodilation, as described above. Improvements in bulk deformability of stored RBCs in AS-1N solutions compared to AS-1 have been demonstrated in previous work, but similar measurements on individual RBCs in hyper- and normoglycemic storage solutions are unprecedented.^11^

A highly promising approach to evaluate the mechanics of individual RBCs is SICM, a noncontact, noninvasive method that has been used to map mechanical properties of soft biological samples.^17,32–34^ A detailed description of the pressurized SICM instrumentation and method described here has been reported.^35^ In a typical SICM setup for mechanical mapping performed here, a nanopipet with a tip size of ∼200 nm outer diameter was filled with PSS buffer, and an Ag/AgCl quasi-reference counter electrode (QRCE) was back-inserted into this pipet. The assembly was then mounted on an XYZ piezo stage. A stepper motor was used to submerge the pipet tip into a bath solution. To prevent contamination of the bath solution with Ag+ ions, the reference electrode was inserted inside a PSS salt bridge. Application of a potential difference, 100 mV, between the two electrodes was used to drive a steady-state ion current through the tip. A constant pressure of 2 kPa was applied at the back of the pipet, creating a nanofluidic flow. The flow exerted force on the sample surface and local sample compliance was revealed through ion current feedback when the pipet was approached to the surface. As shown in Figure 5a, the ion current decreases slower over an RBC (soft sample) compared to a glass substrate (hard surface). The slope of the approach curve between 1% and 2% current reduction was used to evaluate local Young’s modulus with the empirical equation proposed by Rheinlaender et al. as^17^

$$E=\mathrm{AP\_}0(\mathrm{s\_}\infty /s-1{)}^{\wedge }\left(-1\right)$$ 2

where the reference response generated from the substrate slopes, s∞, was derived from the median value of the set of slopes of the ion current approach curves. The response of the RBC sample at each XY pixel was determined using linear regression of the 1% to 2% region of approach curves to generate a sample slope map, s. A (in eq 2) is the geometry dependent empirical parameter derived from COMSOL simulation (COMSOL Multiphysics v6.1). In this experiment, A = 0.1617, details of which have been previously reported.^35^

**Figure 5:**
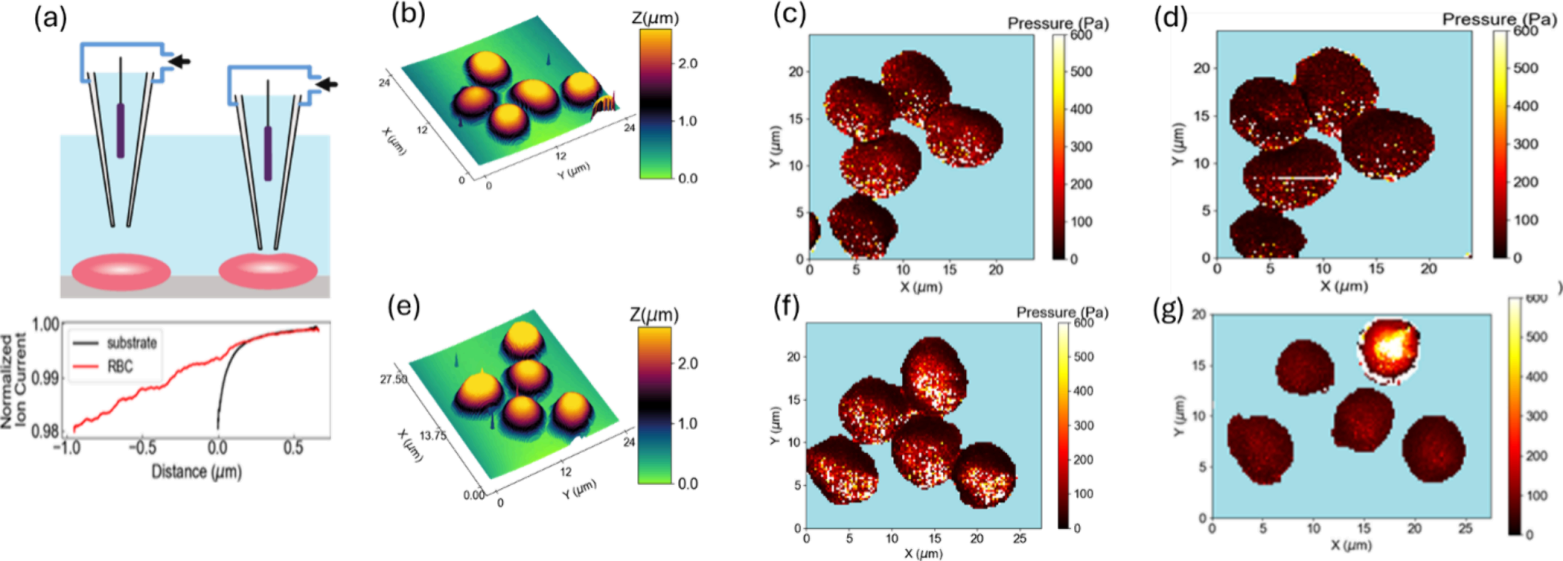
Pressurized-SICM instrumental principle and 3D topographic image and Young’s Modulus image of RBCs using Pressurized-SICM. (a) Local RBC compliance induced by nanofluidic flow from a pressurized nanopipette. The difference in compliance leads to variations in slope of ion current approach curves (shown in (a)). (b) SICM topography of AS-1 stored RBCs in the AS-1 buffer. The corresponding Young’s Modulus images were taken (c) before and (d) after the AS-1 solution was switched to PSS. Similarly, the (e) SICM topography was taken RBCs stored in AS-1N buffer. Young’s Modulus images were scanned at the same region (f) before and (g) after the buffer was switched to PSS. Glass substrate pixels are colored as shallow cyan and are excluded from further statistical analysis.

Interestingly, the data in Figures 5 and 6 suggest that the overall cell rigidity of single cells, reported as the Young’s modulus of each cell, is quite similar for each cell stored in its respective storage solutions, with the AS-1 stored cells exhibiting slightly lower values of Young’s moduli. However, when moved to a plasma-like physiological salt solution, which mimics the actions of an actual transfusion, the AS-1N stored RBCs have measurements of Young’s moduli across the cell surface that indicate an ability to recover cell deformability more readily than the cells stored in the AS-1. For example, in Figures 5c and 5f (RBCs measured while in their AS-1 and AS-1N storage media, respectively) the center moduli are both at ∼300 Pa, which differ from cell edge <100 Pa. When moved to a plasma-like physiological salt solution (PSS), which mimics the actions of an actual transfusion, the moduli of the AS-1 stored RBCs did not change significantly, especially the center moduli. However, the AS-1N stored RBC has measurements of Young’s moduli across the cell surface below 100 Pa (Figure 5g). In summary, these data indicate that while the moduli for the cells in their respective storage solutions were somewhat similar, the moduli of the AS-1N stored cells were able to respond to the media (PSS) change better than the AS-1 stored cells. Interestingly, an examination of AS-1N stored cells after dilution in the PSS suggests that Young’s moduli increased for some of the RBCs (Figure 6, yellow trace) in comparison to the AS-1N cells mapped in AS-1N without buffer exchange in PSS (Figure 6, black trace). It is not confirmed whether this particular “range” of Young’s moduli is favored for increased release of ATP from the RBC upon being subjected to flow.

**Figure 6:**
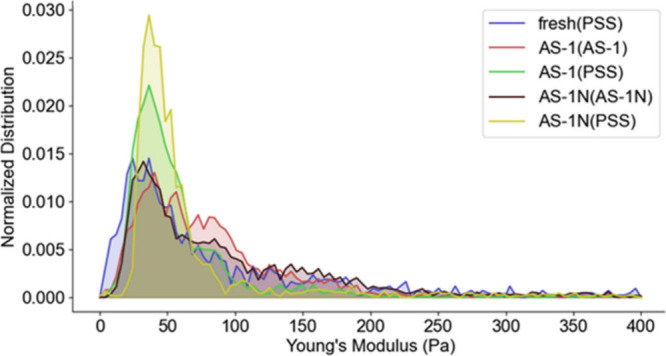
Normalized pixelwise Young’s Modulus distribution of fresh RBC, AS-1 stored RBC in AS-1 imaging medium, AS-1 stored RBC in PSS imaging medium, AS-1N stored RBC in AS-1N imaging medium, and AS-1N stored RBC in PSS imaging medium.

The differences in the release of ATP from the stored RBCs shown in Figure 4, which are often related to cell deformability, coupled with the mapping data suggest that there may be either a specific region of Young’s moduli that are favorable to ATP release or perhaps the AS-1N and AS-1 stored cells have similar moduli but the AS-1 cells have increased glycation damage (as suggested by the glycated hemoglobin data in Figure 3). The Young’s moduli data in Figure 6 are represented as a distribution of the pressure mapping across the RBCs in AS-1, AS-1N, PSS or in AS-1 and AS-1N with buffer exchange.

## Conclusion

Transfusion-transmitted infections, immune-mediated reactions, sepsis, and transfusion-associated circulatory overload (TACO) are all relevant complications that must be considered during a transfusion to a patient recipient.^36,37^ The leading cause of transfusion-related death in the United States, TACO, is caused by rapid transfusion of large volumes leading to hypervolemia or too much fluid in the body. On average, each transfusion recipient will receive two to five units, or 700 to 1750 mL, of RBCs.

Maintaining the therapeutic benefit of transfusion while reducing transfusion volumes would be one way to overcome circulatory overload, but to achieve this goal, an enhancement in transfusion efficacy is needed. Transfusion efficacy is often described as the fraction or percentage of RBCs that remain in circulation 24 h post-transfusion.^38^ Increasing the fraction of intact and circulating RBCs could be attainable by multiple approaches including (1) decreasing the fraction of RBCs that lyse after transfusion, (2) decreasing the fraction of RBCs that are sequestered (e.g., by immune cells or certain organs), or (3) decreasing the fraction of RBCs that adhere to surfaces (such as the endothelium) after transfusion. Unfortunately, the development of detrimental biochemical and physical changes in RBCs throughout storage negatively impacts post-transfusion recovery and clinical outcomes. These changes to the RBC are often collectively referred to as the storage lesion and are associated with damage to proteins and lipids that impair RBC functions that influence post-transfusion outcomes.^39,40^

Here, we report that normoglycemic storage of RBCs using an experimental additive solution can mitigate the development of some of these storage-associated detriments. A novel semiautomated RBC feeding system was developed to maintain normoglycemia throughout storage. Hemolysis, hemoglobin glycation, and flow-induced ATP release were quantified in RBCs stored for up to 8 weeks, and single-cell stiffness was measured after storage for 3 weeks of storage. The performance of the feeding system was validated by frequent blood glucose monitoring, and normoglycemia was well-maintained throughout 8 weeks of storage. Under these conditions, HbA1c and hemolysis were improved near the end of the storage period compared with standard hyperglycemic storage. Significant improvements were seen in flow-induced ATP release from AS-1N stored RBCs throughout storage, indicating superior function relative to their AS-1 stored counterparts. Finally, single-cell measurement of Young’s moduli at the midpoint of storage indicates improved cellular deformability, perhaps contributing to the observed enhanced ATP release. Collectively, these improvements to stored RBCs show that normoglycemic anticoagulant and additive solutions may be beneficial for increasing the efficacy of RBC transfusions. This may have important clinical implications, such as reducing transfusion volumes, which could prevent the onset of serious complications. In addition, this work shows that new analytical tools make it possible to gain insights into the RBC function. Limitations to the current study are primarily focused on the lack of a noninvasive method to monitor the glucose concentrations in the bags throughout storage. Overcoming this limitation, without having to invade the bag or sample from the bag, would enable the glucose concentrations to be controlled within a set range by having on-demand feeding within minutes of checking the glucose levels.

## Supplementary Material



## Notes

Details regarding control server, software, and networking for glucose feeding system (PDF)

## References

[1] Baskett T. F. (2002). James Blundell:
the first transfusion of human blood. Resuscitation.

[2] Free R. J., Sapiano M. R. P., Chavez Ortiz J. L., Stewart P., Berger J., Basavaraju S. V. (2023). Continued stabilization of blood collections and transfusions
in the United States: Findings from the 2021 National Blood Collection
and Utilization Survey. Transfusion.

[3] Jahr J. S., Guinn N. R., Lowery D. R., Shore-Lesserson L., Shander A. (2021). Blood Substitutes and
Oxygen Therapeutics: A Review. Anesth Analg.

[4] Alayash A. I. (2014). Blood substitutes:
why haven’t we been more successful?. Trends Biotechnol.

[5] Palmer A. F., Intaglietta M. (2014). Blood substitutes. Annu. Rev.
Biomed Eng..

[6] Current Good Manufacturing Practice for Blood and Blood Components. Code of Federal Regulations, Title 21 § 606. www.ecfr.gov/current/title-21/chapter-I/subchapter-F/part-606. Accessed July 23, 2024.

[7] Requirements for Blood and Blood Components Intended for Transfusion or for Further Manufacturing Use. Code of Federal Regulations, Title 21 § 630. www.ecfr.gov/current/title-21/chapter-I/subchapter-F/part-630. Accessed July 16, 2024.

[8] Additional Standards for Human Blood and Blood Products. Code of Federal Regulations, Title 21 § 640. www.ecfr.gov/current/title-21/chapter-I/subchapter-F/part-640. Accessed July 16, 2024.

[9] Hess J. R. (2006). An update
on solutions for red cell storage. Vox Sang.

[10] Brecher, M. E. ; Linden, J. V. ; Roseff, S. D. Collection, Preparation, Storage, and Distribution of Components from Whole Blood Donations, 15th ed.; Association for the Advancement of Blood & Biotherapies: 2005; pp 175–202.

[11] Liu Y., Hesse L. E., Geiger M. K., Zinn K. R., McMahon T. J., Chen C., Spence D. M. (2022). A 3D-printed transfusion platform
reveals beneficial effects of normoglycemic erythrocyte storage solutions
and a novel rejuvenating solution. Lab Chip.

[12] Mu R. P., Chen C. P., Wang Y. M., Spence D. M. (2016). A quantitative,
appraisal of experimental low-glucose storage solutions used for blood
banking. Anal Methods-Uk.

[13] Wang Y., Giebink A., Spence D. M. (2014). Microfluidic
evaluation of red cells
collected and stored in modified processing solutions used in blood
banking. Integr Biol. (Camb).

[14] Soule L. D., Skrajewski-Schuler L., Branch S. A., McMahon T. J., Spence D. M. (2024). Toward
Translational Impact of Low-Glucose Strategies on Red Blood Cell Storage
Optimization. ACS Pharmacol Transl Sci..

[15] Hayter E. A., Azibere S., Skrajewski L. A., Soule L. D., Spence D. M., Martin R. S. (2022). A 3D-printed, multi-modal microfluidic device for measuring
nitric oxide and ATP release from flowing red blood cells. Anal Methods.

[16] Hayter E. A., Castiaux A. D., Martin R. S. (2020). 3D-Printed
Microfluidic Device with
In-line Amperometric Detection that Also Enables Multi-Modal Detection. Anal Methods.

[17] Rheinlaender J., Schäffer T. E. (2013). Mapping
the mechanical stiffness of live cells with
the scanning ion conductance microscope. Soft
Matter.

[18] Moore G. L. (1987). Additive
solutions for better blood preservation. Crit
Rev. Clin Lab Sci..

[19] Livshits L., Barshtein G., Arbell D., Gural A., Levin C., Guizouarn H. (2021). Do We Store Packed Red Blood Cells
under ″Quasi-Diabetic″
Conditions?. Biomolecules.

[20] D’Alessandro A., Mirasole C., Zolla L. (2013). Haemoglobin
glycation (Hb1Ac) increases
during red blood cell storage: a MALDI-TOF mass-spectrometry-based
investigation. Vox Sang.

[21] Prosenz J., Ohlinger T., Mullner E. W., Marculescu R., Gerner C., Salzer U., Kiefer F. W., Baron D. M. (2019). Glycated
hemoglobin concentrations of red blood cells minimally increase during
storage under standard blood banking conditions. Transfusion.

[22] Szelenyi J. G., Foldi J., Hollan S. R. (1983). Enhanced nonenzymatic glycosylation
of blood proteins in stored blood. Transfusion.

[23] Zeller W. P., Eyzaguirre M., Hannigan J., Ozog K., Suarez C., Silberman S., Hoffstadter A., Hurley R. M. (1985). Fast hemoglobins
and red blood cell metabolites in citrate phosphate dextrose adenine
stored blood. Ann. Clin Lab Sci..

[24] Faris A., Spence D. M. (2008). Measuring the simultaneous
effects of hypoxia and deformation
on ATP release from erythrocytes. Analyst.

[25] Price A. K., Fischer D. J., Martin R. S., Spence D. M. (2004). Deformation-induced
release of ATP from erythrocytes in a poly­(dimethylsiloxane)-based
microchip with channels that mimic resistance vessels. Anal. Chem..

[26] Sprague R. S., Ellsworth M. L., Stephenson A. H., Kleinhenz M. E., Lonigro A. J. (1998). Deformation-induced
ATP release from red blood cells
requires CFTR activity. Am. J. Physiol..

[27] Wan J., Ristenpart W. D., Stone H. A. (2008). Dynamics of shear-induced ATP release
from red blood cells. Proc. Natl. Acad. Sci.
U. S. A..

[28] Spence D. M., Torrence N. J., Kovarik M. L., Martin R. S. (2004). Amperometric determination
of nitric oxide derived from pulmonary artery endothelial cells immobilized
in a microchip channel. Analyst.

[29] Chen C. P., Wang Y. M., Lockwood S. Y., Spence D. M. (2014). 3D-printed fluidic
devices enable quantitative evaluation of blood components in modified
storage solutions for use in transfusion medicine. Analyst.

[30] Qiang Y., Sissoko A., Liu Z. L., Dong T., Zheng F., Kong F., Higgins J. M., Karniadakis G. E., Buffet P. A., Suresh S., Dao M. (2023). Microfluidic
study
of retention and elimination of abnormal red blood cells by human
spleen with implications for sickle cell disease. Proc. Natl. Acad. Sci. U. S. A..

[31] McNamee A. P., Simmonds M. J. (2023). Red Blood Cell Sublethal
Damage: Hemocompatibility
Is not the Absence of Hemolysis. Transfus Med.
Rev..

[32] Hansma P. K., Drake B., Marti O., Gould S. A., Prater C. B. (1989). The scanning
ion-conductance microscope. Science.

[33] Sanchez D., Johnson N., Li C., Novak P., Rheinlaender J., Zhang Y., Anand U., Anand P., Gorelik J., Frolenkov G. I., Benham C., Lab M., Ostanin V. P., Schaffer T. E., Klenerman D., Korchev Y. E. (2008). Noncontact measurement
of the local mechanical properties of living cells using pressure
applied via a pipette. Biophys. J..

[34] Zhu C., Huang K., Siepser N. P., Baker L. A. (2021). Scanning Ion Conductance
Microscopy. Chem. Rev..

[35] Wang Y., Shashishekar M., Spence D. M., Baker L. A. (2025). Subcellular Mechanical
Imaging of Erythrocytes with Optically Correlated Scanning Ion Conductance
Microscopy. ACS Measurement Science Au.

[36] Raval J. S., Griggs J. R., Fleg A. (2020). Blood Product
Transfusion in Adults:
Indications, Adverse Reactions, and Modifications. Am. Fam Physician.

[37] Sharma S., Sharma P., Tyler L. N. (2011). Transfusion of blood and blood products:
indications and complications. Am. Fam Physician.

[38] Hess J. R. (2012). Biomedical
Excellence for Safer Transfusion, C., Scientific problems in the regulation
of red blood cell products. Transfusion.

[39] Luten M., Roerdinkholder-Stoelwinder B., Schaap N. P., de Grip W. J., Bos H. J., Bosman G. J. (2008). Survival of red blood cells after
transfusion: a comparison between red cells concentrates of different
storage periods. Transfusion.

[40] Yoshida T., Prudent M., D’Alessandro A. (2019). Red blood
cell storage lesion: causes
and potential clinical consequences. Blood Transfus.

