# Supplementary material for: Microfluidic Determination of Cell-Derived ATP and Single Cell Pressure Mapping Confirms Benefits of Normoglycemic Stored Red Blood Cells
Source: ACS Meas Sci Au. 2025 Jul 3;5(4):511–9. doi: 10.1021/acsmeasuresciau.5c00032 (PMC12371595; doi:10.1021/acsmeasuresciau.5c00032)
Supplement: Supplementary file 1 [file tg5c00032_si_001.pdf]

Supplemental Information to Accompany

Microfluidic Determination of Cell-derived ATP and Single Cell Pressure Mapping Confirms Benefits of Normoglycemic Stored Red Blood Cells

*Stephen A. Branch[1,3], Yunong Wang[6], Samuel Azibere[5], Logan D. Soule[1,3], Ashley R. Davis[1,3], Timothy McMahon[4], R. Scott Martin[5], Lane A. Baker[6], Morgan K. Geiger[1,3], and Dana M. Spence[1,3]**

Departments of Biomedical Engineering[1], Chemistry[2], and Institute for Quantitative Health Science and Engineering[3], Michigan State University, East Lansing, MI 48824, USA

Department of Medicine[4], Duke University, Durham, NC 27710, USA

Department of Chemistry[5], Saint Louis University, St. Louis, MO 63103, USA

Department of Chemistry[6], Texas A & M University, College Station, TX 77843, USA

Corresponding author*:
Dana M. Spence
spenceda@msu.edu
(517) 353-1116
775 Woodlot Dr.
East Lansing, MI 48824

The authors report no conflicts of interest with this work.

The authors acknowledge NHLBI (HL156440) for research support.

All data will be made available upon request to the authors.

Control Server. The first-generation feeding system utilized an open-source single-board microcontroller (SBM) (Arduino Uno R3) to actuate the solenoid valve. These development boards are inexpensive, simple to work with and operate, and allow for rapid prototyping when interfacing with hardware. For these reasons, it was decided the second-generation feeding system would also use this platform for controlling the peristaltic pumps. Unfortunately, these SBMs have insufficient computing power for the planned software upgrades. Thus, an open-source single-board computer (SBC) (Orange Pi Zero2) was selected to act as a control server. This SBC can run the software and communicate commands to the SBM, enabling simple hardware control with sophisticated software. The SBC also is capable of acting as a wireless access point, allowing easier access to the user interface.

Software. The SBC software can be broken down into four modules, each handling a separate set of functions for the application. This software runs in a virtualized container on the Docker platform, enabling easy deployment and upgrades. It also takes advantage of container restart policies to ensure the application is always running, even after an SBC reboot or power failure. The entirety of the application is written in the Python programming language.[1]

The first module runs the control loop that calculates the volume of feeding solution to be dispensed into each bag. This control loop is executed at a user-defined interval; for these studies, it was run every five minutes. For each bag currently in the system, individual bag parameters are calculated for maximum dosing accuracy and experimental flexibility. Figure S1 shows a graphical overview of the control loop logic and calculations.

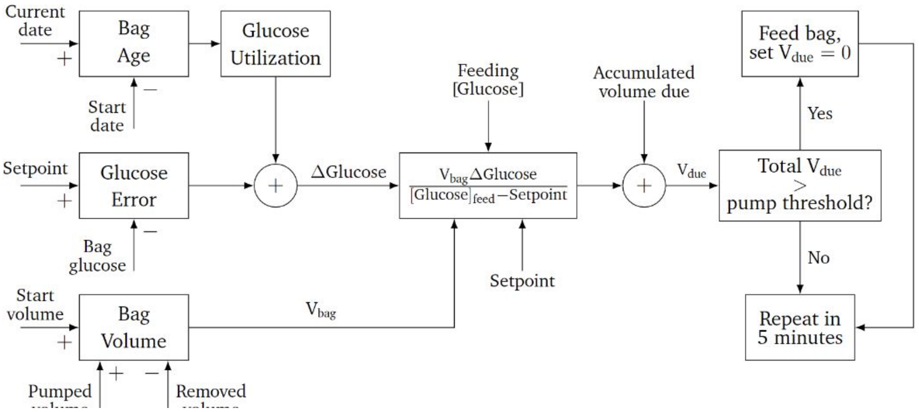

Figure S1. Block diagram of the algorithm used by the control loop of the semi-autonomous feeding system. This calculates the volume of feeding solution to be delivered to each bag every five minutes. Bag parameters are tracked individually for "personalized" feeding, which occurs when the feed volume exceeds the pumping threshold.

The second module handles object-relational mapping to read and write from a persistent database. For this application, an SQLite database is used to store application settings and experimental data.[2] This is a popular embedded database that does not require a separate database server. The database is stored as a single file outside of the application container, allowing for simple data backups and restorations. Other modules of the application use an object-oriented approach to handling data; this module serves as a translator to read and write the objects to and from the database.

The third module handles asynchronous communications between the SBC and SBM. This is accomplished via serial communication over a Universal Serial Bus (USB) cable. The control server sends commands over USB, which are acted upon by the SBM. A timestamped return communication to the control server is logged in the database indicating if the command was successfully executed. The fourth module is a web user interface for managing the bags being stored, written with the NiceGUI Python package.[3] This displays the status of each blood bag currently in storage, as well as any data associated with them. An archive of previously stored blood bags is also available, as is the ability to download a copy of the database. Individual pump settings are available on another page of the interface, along with manual pump control options for calibration and priming. Finally, an error log is available to view any exceptions that occur in the application. Figure S2 shows what the home screen of this interface may look like with four bags in storage.

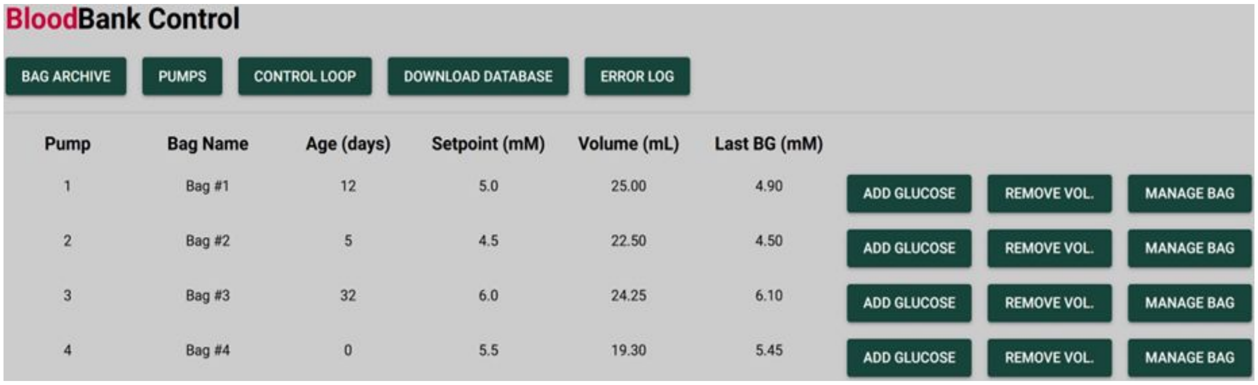

Figure S2. Home screen of the web user interface for the blood banking software. An overview of bags currently in storage is shown. Buttons link to other pages of the interface.

Networking. In order to connect to the web user interface hosted on the control server, clients must be connected to the same network as the SBC. Fortunately, the SBC can

easily be configured to act as a Wi-Fi host, broadcasting its own network to other devices. This negates the need to connect the SBC and client devices to an uncontrolled third-party network and simplifies communications between them. The tradeoff of this approach is the additional networking configuration required for the initial setup.

To this end, the dnsmasq software package is used.[4] This provides lightweight and easily configurable Domain Name System (DNS) and Dynamic Host Configuration Protocol (DHCP) servers that can run on the SBC. Briefly, the DHCP server assigns an Internet Protocol (IP) address to the client device and, among other information, provides IP addresses the client should use for DNS requests. The DHCP server used here is configured to provide the IP address of the SBC itself as the DNS server.

DNS servers translate human-readable website domain names to the IP addresses used by computers. Thus, a client device connected to the SBC Wi-Fi will request the IP address of a website from the SBC itself. Here, the DNS server is configured to provide the IP address of the blood banking software web server in response to any query. In other words, when connected to the SBC network, navigating to any website in a web browser will redirect you to the application interface where the user can control parameters to maintain glucose concentrations.

LITERATURE CITED

(1) Python Software Foundation. Python. Version 3.12. https://www.python.org/.

(2) D. Richard Hipp. SQLite. Version 3.43.2. https://www.sqlite.org/.

(3) Zauberzeug GmbH. NiceGUI. Version 1.3.15. https://nicegui.io/.

(4) Simon Kelley. Dnsmasq. Version 2.89. https://dnsmasq.org/doc.html.
